# Supplementary material for: Comparative genomics of smut fungi suggest the ability of meiosis and mating in asexual species of the genus Pseudozyma (Ustilaginales)
Source: BMC Genomics. 2023 Jun 13;24:321. doi: 10.1186/s12864-023-09387-1 (PMC10262431; doi:10.1186/s12864-023-09387-1)
Supplement: Supplementary file 1 — Additional file 1: Supplementary Figure 1. Phylogenetic tree of translated PRA gene sequences. The tree shows three clear clades of receptor gene alleles (one allele per species), allowing the annotation of mating-types for each strain. [file 12864_2023_9387_MOESM1_ESM.pdf]

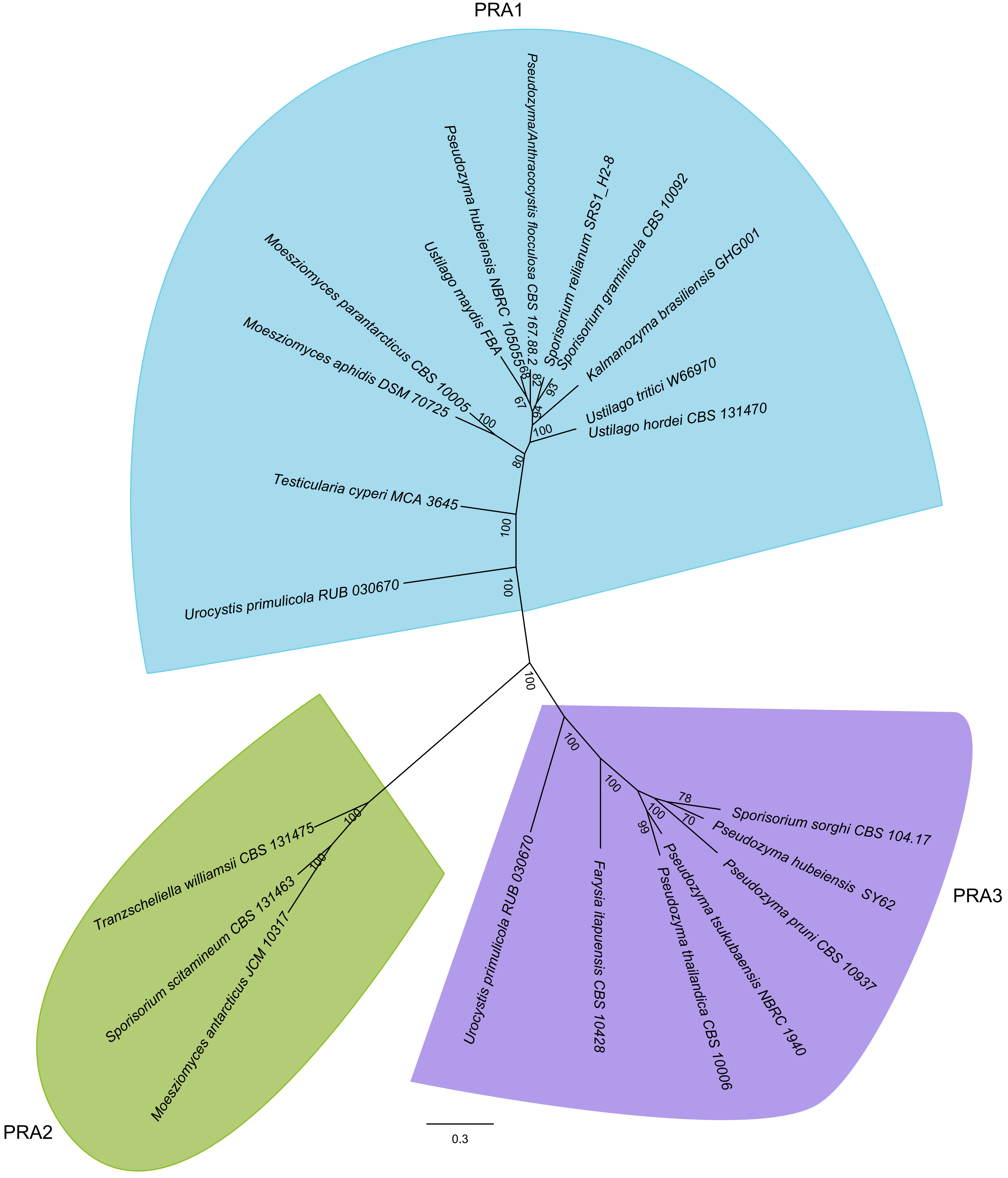

Supplementary Figure 1:
Phylogenetic tree of translated PRA gene sequences. The tree shows three clear clades of receptor gene alleles (one allele per species), allowing the annotation of mating-types for each strain.
