# Supplementary material for: Comparative genomics of smut fungi suggest the ability of meiosis and mating in asexual species of the genus Pseudozyma (Ustilaginales)
Source: BMC Genomics. 2023 Jun 13;24:321. doi: 10.1186/s12864-023-09387-1 (PMC10262431; doi:10.1186/s12864-023-09387-1)
Supplement: Supplementary file 2 — Additional file 2: Supplementary Figure 2. Phylogenetic tree of translated MFa gene sequences. The tree shows three clear clades of pheromone gene alleles (1-2 alleles per species, allowing the annotation of pheromones to the mating-types of the strains and establishing self-sterility for Ustilaginales. Support values >50 are indicated with a dot, pseudogenized pheromones are shown in grey. Pheromone nomenclature follows compatibility, with MFaX.1 being compatible to PRA1, MFaX.2 being compatible to PRA2, and MFaX.3 being compatible to PRA3. [file 12864_2023_9387_MOESM2_ESM.pdf]

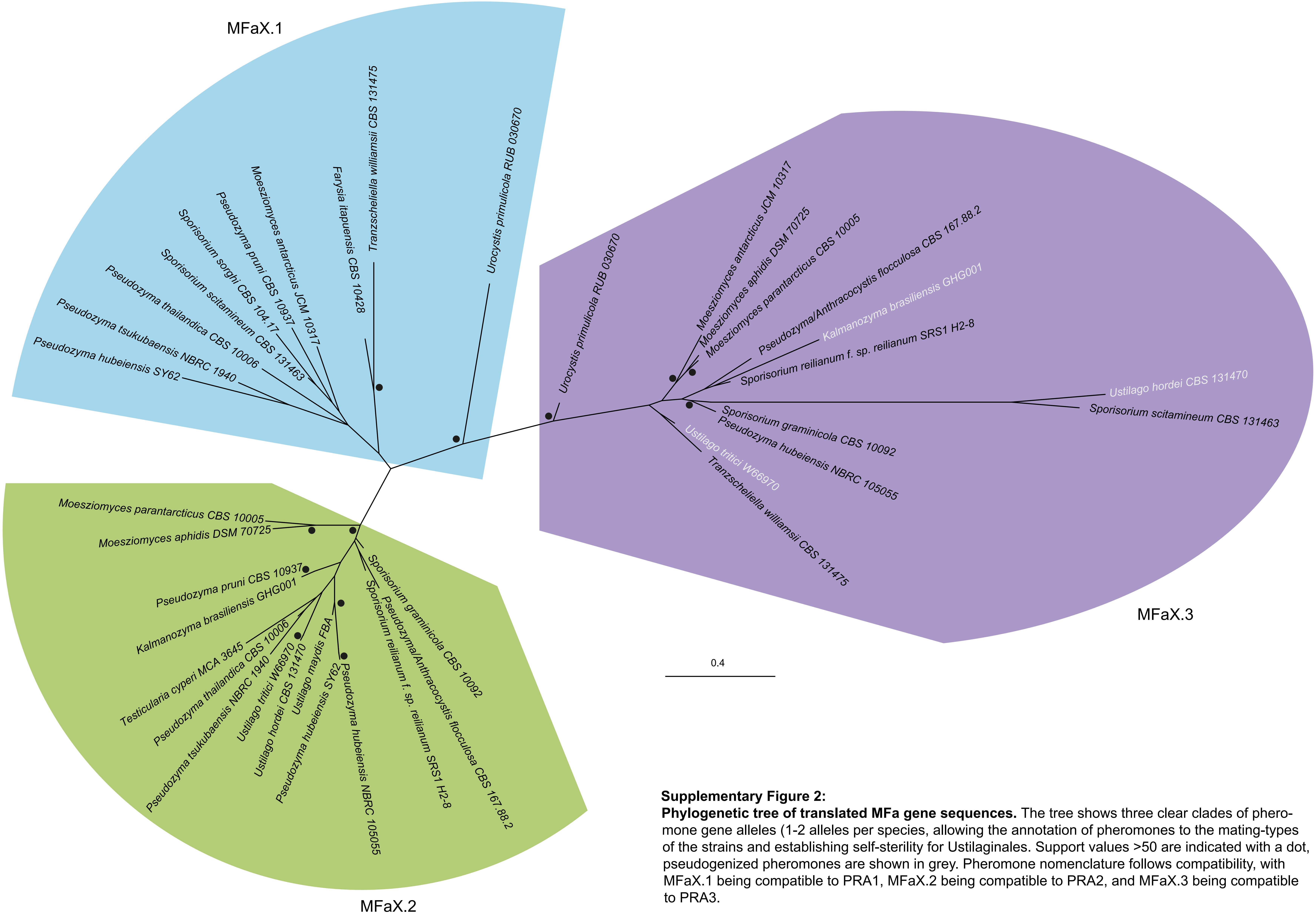


**Supplementary Figure 2:**
**Phylogenetic tree of translated MFa gene sequences.** The tree shows three clear clades of pheromone gene alleles (1-2 alleles per species, allowing the annotation of pheromones to the mating-types of the strains and establishing self-sterility for Ustilaginales. Support values >50 are indicated with a dot, pseudogenized pheromones are shown in grey. Pheromone nomenclature follows compatibility, with MFaX.1 being compatible to PRA1, MFaX.2 being compatible to PRA2, and MFaX.3 being compatible to PRA3.
