# Supplementary figures and images for: Comparative genomics of smut fungi suggest the ability of meiosis and mating in asexual species of the genus Pseudozyma (Ustilaginales)
Source: BMC Genomics. 2023 Jun 13;24:321. doi: 10.1186/s12864-023-09387-1 (PMC10262431; doi:10.1186/s12864-023-09387-1)

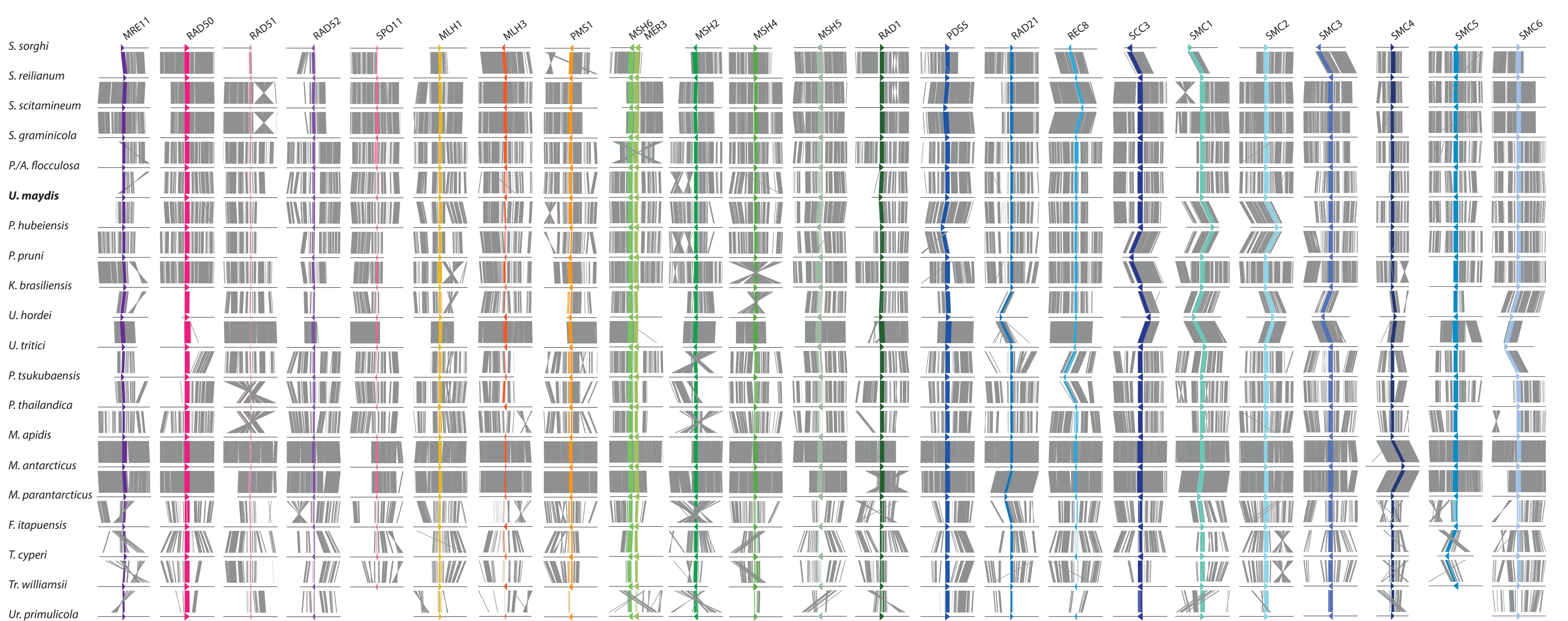

10kb

Supplement: Supplementary file 3 — Additional file 3: Supplementary Figure 3. Synteny of core meiosis gene loci. Annotated genes and flanking regions (20 kb) remain mostly syntenic in relatively closely related species. More distantly related species like Tr. williamsii, T. cyperi, and the outgroup Ur. primulicola show less synteny. Rearrangements in the genomes of Pseudozyma compared to the sexual species cannot be noted. Species are sorted according to relatedness in the phylogenomic tree. [file 12864_2023_9387_MOESM3_ESM.pdf]
